# Differencing strategies for SLR observations at the Wettzell observatory

**DOI:** 10.1007/s00190-021-01588-4

**Published:** 2021-12-25

**Authors:** Iván Herrera Pinzón, Markus Rothacher, Stefan Riepl

**Affiliations:** 1 Institute of Geodesy and Photogrammetry, ETH Zurich, Zurich, Switzerland; 2 Federal Agency for Cartography and Geodesy, Wettzell Observatory, Leipzig, Germany

**Keywords:** Satellite laser ranging, Single and double differences, Local ties

## Abstract

The precise estimation of geodetic parameters using single- and double-differenced SLR observations is investigated. While the differencing of observables is a standard approach for the GNSS processing, double differences of simultaneous SLR observations are practically impossible to obtain due to the SLR basic principle of observing one satellite at a time. Despite this, the availability of co-located SLR telescopes and the use of the alternative concept of *quasi-simultaneity* allow the forming of SLR differences under certain assumptions, thus enabling the use of these processing strategies. These differences are in principle almost free of both, satellite- and station-specific error sources, and are shown to be a valuable tool to obtain relative coordinates and range biases, and to validate local ties. Tested with the two co-located SLR telescopes at the Geodetic Observatory Wettzell (Germany) using SLR observations to GLONASS and LAGEOS, the developed differencing approach shows that it is possible to obtain single- and double-difference residuals at the millimetre level, and that it is possible to estimate parameters, such as range biases at the stations and the local baseline vector with a precision at the millimetre level and an accuracy comparable to traditional terrestrial survey methods. The presented SLR differences constitute a valuable alternative for the monitoring of the local baselines and the estimation of geodetic parameters.

## Introduction

Satellite laser ranging (SLR) is one of the four main geodetic techniques involved in the realisation of the International Terrestrial Reference Frame (ITRF). In particular, SLR contributes to the determination of the scale and the origin of the frame, but it is also involved in the determination of the gravity field of the Earth, and it is widely used for the validation of satellite orbits. In the operating principle of SLR, at an SLR ground station, a short laser pulse is generated and sent with an optical telescope to a single satellite. The satellite has retro-reflectors attached to its surface that reflect the laser pulse back to the ground station. With the telescope at the ground station the reflected pulse is then detected and analysed. Based on the time of emission of the original pulse and the time of reception of the reflected pulse, the light travel time, the range between the satellite and the telescope can be computed. Core stations of the International Laser Ranging Service (ILRS) (Pearlman et al. 2019b) are able to provide normal point measurements with a millimetre-level precision (Luceri et al. 2019). Thus, observations to SLR-dedicated satellite missions, such as LAGEOS or Etalon, and more recently to Global Navigation Satellite Systems (GNSS) satellites, turn SLR into a reliable and accurate technique for the determination of geodetic and geophysical parameters. However, systematic errors at the station level together with mismodellings of the centre of masses of the satellites, threaten the accuracy of these range observations. To this end, the ILRS performs the continuous monitoring of systematic error sources at the station level, for an enhancement of the realisation of the ITRF, searching for improving the agreement between the scale provided by SLR and the remaining geodetic techniques (Luceri et al. 2019). Systematic time biases in range observations (which amount to few $\mu$s) degrade the accuracy of the estimated satellite orbits, affecting the station coordinates by several millimetres, and have to be removed, for instance with the time synchronisation of stations using time transfer by laser links to satellites (Exertier et al. 2017).

At the Geodetic Observatory Wettzell, two SLR telescopes, increasing the observation capabilities, have been operating for quite some time. In consequence, an ideal setup for the assessment and testing of the differencing approach has become available on a very short SLR baseline. The two co-located SLR telescopes, connected by a local tie, controlled by a common timing system and affected by (almost) the same atmospheric effects, allow the study of the size and stability of instrumental biases, the quality of the local tie, and a detailed investigation of new processing strategies. Forming double differences is a standard approach in GNSS processing (Hofmann-Wellenhof et al. 2008). The advantage of forming single or double differences is the elimination (or strong reduction) of satellite- (differences between stations) and station-specific (differences between satellites) error sources and the high accuracy achieved for short baselines, as well as an adequate approach to reduce the number of parameters (especially clock corrections) during the estimation procedure. Applying these methods to SLR observations results in a tool to assess existing satellite- and station-specific biases in SLR, and to estimate accurate relative coordinates between neighbouring telescopes, an important approach to validate local ties. However, it is very difficult to obtain simultaneous SLR observations in practice. While two SLR telescopes can in principle observe the same satellite simultaneously, when given the same schedule for the observation and under optimal weather conditions, simultaneous SLR observations from one telescope to two satellites are not feasible in practice. Therefore simultaneous double differences cannot be formed, and therefore, we have to resort to *quasi-simultaneous* observations.

The idea of *quasi-simultaneity* was first introduced in Pavlis (1985) as a tool to avoid the propagation of orbital errors, implicitly included in the range observations, into the estimated parameters. To achieve simultaneous SLR observations, the ranges of two stations were interpolated using cubic splines. Based on a large set of observations to the LAGEOS satellites, as well as multiple simulations, his thorough analysis showed that the use of differenced ranges mitigates orbital errors and achieves the estimation of baseline lengths with centimetre-level accuracy, even under the presence of satellite orbit errors on the metre level. Additionally, the quality of this method was demonstrated to be related to the geometry of the network and the observed satellites, with the best results obtained from passes parallel to the baseline direction. Similarly, Dedes and Müller (1989) used the idea of quasi-simultaneous observations from pairs of stations to obtain simultaneous range differences for the estimation of baselines independently of orbital errors. Their work used SLR observations to LAGEOS satellites, and the quasi-simultaneity is achieved through the interpolation of the observed laser ranges with Chebyshev polynomials, and a careful procedure for the elimination of outlier observations. They concluded that, given enough observations, it is possible to estimate baselines up to 1000 km with centimetre-level accuracy. A concept for double differences of SLR observations was later explored by Svehla (2018) with the baseline between the telescopes GRZL at Graz (Austria) and HERL at Herstmonceux (UK), and observing only two Galileo satellites. This approach forms SLR normal points at common epochs for both satellites, using first- order polynomials fitted to the normal points of the GRZL station, so that its SLR normal points are interpolated to epochs of the normal points of the HERL station, separately for each satellite and tracking pass. By fixing the IGS orbits, his study shows that the formed double differences allow the estimation of relative coordinates for the baseline GRZL-HERL at the level of 1-8 mm.

In contrast, our approach for building differences uses the advantage of a very short SLR baseline, with a custom-made quasi-simultaneity strategy to build single and double differences based on the linearised observation equations derived for the original ranges, without the need to interpolate either the normal points nor the original ranges. We only require a continuous representation of the satellite orbits, i.e. dynamic orbits. Additionally we try to maximise the number of observations by focusing on more than two satellites simultaneously, including SLR observations to both, GNSS and LEO satellites. From this perspective, this paper discusses the applicability and potential of the differencing approaches, namely single and double differences, for the estimation of geodetic parameters with SLR observations. These differences, together with the original ranges (zero differences), are used to get estimates of both, satellite- and station-specific error sources, so that systematic effects common to both stations can be identified at millimetre level. This approach, therefore, may potentially improve the processing of classical SLR observations of GNSS and LEO satellites and the estimation of accurate local ties. The rest of the paper is structured as follows: Sect. 2 outlines the concept of quasi-simultaneity, which constitutes the basis of our work. The available data set and the processing strategy used for the estimation of parameters are described in Sect. 3. Before summarising the paper in Sect. 5, Sect. 4 discusses the results derived from the short SLR baseline in Wettzell regarding station coordinates and range biases, and the comparison of the derived baseline vector with the terrestrial local tie.

**Fig. 1:**
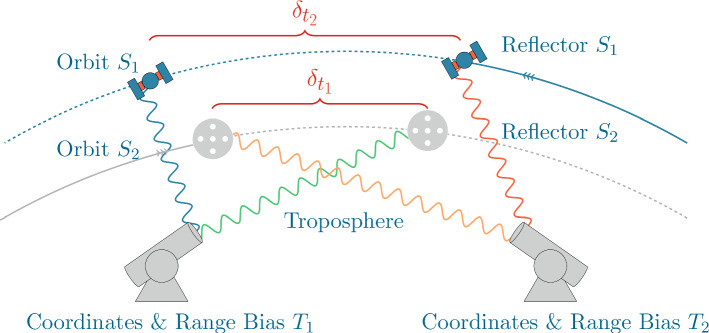
Concept of quasi-simultaneity for the differencing of SLR observations, together with the error sources targeted with these approaches. Two satellites ($S_1$ and $S_2$) in different orbits are observed. The quasi-simultaneity of the observations to satellite $S_1$ is given by $\delta _{t_1}$, while the quasi-simultaneity of the observations to satellite $S_2$ is given by $\delta _{t_2}$

## Idea and formalism

### Concept of quasi-simultaneity

The fundamental tool to build single and double differences of SLR observations is the concept of *quasi-simultaneous* observations. Two observations are considered quasi-simultaneous if they lie within a specified time window. This is, range *i* from telescope 1 to satellite *k* observed at time $t_i$ ($\rho _{1}^{k}(t_i)$) and range *j* from telescope 2 to satellite *l* at time $t_j$ ($\rho _{2}^{l}(t_j)$) are considered quasi-simultaneous if $\Vert (t_j - t_i)\Vert \le \delta _t$, where $\delta _t$ is the so-called quasi-simultaneity, a fixed value. If $t_j$ and $t_i$ satisfy this condition, they are considered *quasi-simultaneous epochs*. Figure 1 shows the concept of quasi-simultaneity for an SLR baseline, where the time epochs for the observations from telescope 2 with respect to telescope 1 are $t_1$ and $t_2$ for satellite 1 and 2, respectively. Using this idea, three types of differences can be built, namely

1. Single differences from two telescopes to the same satellite:
  $$\begin{aligned} SD_{1,2}^{l}(t_i,t_j) = \rho _{2}^{l}(t_j) - \rho _{1}^{l}(t_i),\ \ \ \mathrm {for}\ \ \ \Vert (t_j - t_i)\Vert \le \delta _t \end{aligned}$$
  By observing the same satellite from two telescopes at quasi-simultaneous epochs, orbit and retro-reflector errors are strongly reduced. For the short baseline in Wettzell the tropospheric delays are eliminated as well (apart from the effect of the height difference between the telescopes).
2. Single differences from one telescope to two satellites:
  $$\begin{aligned} SD_{m}^{k,l}(t_i,t_j)= & {} \rho _{m}^{l}(t_j) - \rho _{m}^{k}(t_i),\\&\mathrm {for}\ \ \ \Vert (t_j - t_i)\Vert \le \delta _t,\ \ \ m=1,2 \end{aligned}$$
  This definition includes the special case of observing the same satellite at two different epochs, as long as the quasi-simultaneity condition is met:
  $$\begin{aligned} SD_{m}^{k}(t_i,t_j)= & {} \rho _{m}^{k}(t_j) - \rho _{m}^{k}(t_i),\\&\text {for}\ \ \ \Vert (t_j - t_i)\Vert \le \delta _t,\ \ \ m=1,2 \end{aligned}$$
  If observations are made from one telescope to two satellites at quasi-simultaneous epochs, the influence of instrumental biases caused by the station setup is practically eliminated.
3. Double differences:
  $$\begin{aligned}&DD_{1,2}^{k,l}(t_{i_1},t_{j_1},t_{i_2},t_{j_2}) \\&\quad = \left( \rho _{2}^{l}(t_{j_1}) - \rho _{1}^{l}(t_{i_1})\right) - \left( \rho _{2}^{k}(t_{j_2}) - \rho _{1}^{k}(t_{i_2})\right) , \end{aligned}$$
  for $\Vert (t_{j_1} - t_{i_1})\Vert \le \delta _{t_1}$ and $\Vert (t_{j_2} - t_{i_2})\Vert \le \delta _{t_2}$. Similarly to the single differences from one telescope to two satellites, building double differences with the concept of quasi-simultaneous observations allows the special case of having double differences to the same satellite ($k=l$) at different epochs. Double differences are expected to eliminate both, satellite-specific (orbits and retro-reflectors) and station-specific (instrumental biases) errors.

### Single-difference observation equation from two telescopes to one satellite

From a practical perspective, the SLR ranges are processed as in a usual SLR processing to obtain the so-called *zero-difference* linearised observation equations. These zero-difference linearised observation equations are later differenced to obtain the single- and double-difference systems of linear equations. The use of zero difference ensures that the original ranges are processed at the exact epoch, which in turn avoids the necessity to interpolate the two ranges to a common epoch. Formally, given an observation from the telescope *m* to the satellite *k* at time $t_i$, $\rho _{m}^{k}(t_i)$, the simplified SLR observation equation reads as

$$\begin{aligned} \rho _{m}^{k}(t_i) = \mathrm {P}_{m}^{k} + \delta \rho _{m,atm}^k + \delta \rho _{m,rel}^k + \delta \rho _{m,sys} + \delta \rho _{sys}^k + \varepsilon _{m}^{k} \end{aligned}$$

with $\mathrm {P}_{m}^{k}$ the geometrical distance between the satellite and the station at the observation time $t_i$, $\delta \rho _{m,atm}^k$ the delay (refraction) in the troposphere, $\delta \rho _{m,rel}^k$ the relativistic correction, $\delta \rho _{m,sys}$ delays in the laser system (among others a range bias $B_m$), $\delta \rho _{sys}^k$ the retroreflector correction and $\varepsilon _{m}^{k}$ the measurement error. Using standard models to account for the troposphere, such as Mendes and Pavlis (2004), applying the relativistic correction, and using precise orbits for the satellites, the linearised zero-difference observation equations are given by

$$\begin{aligned} {\mathbf {A}}_{m}^{ZD}\,\varvec{\Delta }{\mathbf{x}} - {\mathbf {b}}_{m}^{ZD} = {\mathbf {v}}_m^{ZD} \end{aligned}$$

where $\varvec{\Delta }\mathbf {x}=(x_m, y_m, z_m, B_m)^T$ is the vector of the unknown parameters containing geocentric coordinates and range biases, ${\mathbf {A}}_m^{ZD}$ contains the partials of the observation equation with respect to the unknowns

$$\begin{aligned} \begin{pmatrix} \dfrac{\partial \rho _{m}^{k}(t_i)}{\partial x_k(t_i)},&\dfrac{\partial \rho _{m}^{k}(t_i)}{\partial y_k(t_i)},&\dfrac{\partial \rho _{m}^{k}(t_i)}{\partial z_k(t_i)},&\dfrac{\partial \rho _{m}^{k}(t_i)}{\partial B_k(z_i)} \end{pmatrix} \end{aligned}$$

and each element of the reduced observation vector (observed–computed) ${\mathbf {b}}_{m}^{ZD} = [b_{m}^{k}(t_i)]$ is given by

$$\begin{aligned} b_{m}^{k}(t_i) = \rho _{m}^{k}(t_i) - {\rho _0}_m^k(t_i) \end{aligned}$$

with ${\rho _0}_m^k(t_i)$ the result of the a-priori values applied in the observation equation. When $n_1$ and $n_2$ observations are available for telescope 1 and 2, respectively, the system of linear equations is given by

$$\begin{aligned}&\mathbf {A_{m}^{ZD}} = \begin{bmatrix} \dfrac{\partial \rho _{m}^{1}(t_1)}{\partial x_1(t_1)} &{} \dfrac{\partial \rho _{m}^{1}(t_1)}{\partial y_1(t_1)} &{} \dfrac{\partial \rho _{m}^{1}(t_1)}{\partial z_1(t_1)} &{} \dfrac{\partial \rho _{m}^{1}(t_1)}{\partial B_1(t_1)}\\ \dfrac{\partial \rho _{m}^{2}(t_2)}{\partial x_2(t_2)} &{} \dfrac{\partial \rho _{m}^{2}(t_2)}{\partial y_2(t_2)} &{} \dfrac{\partial \rho _{m}^{2}(t_2)}{\partial z_2(t_2)} &{} \dfrac{\partial \rho _{m}^{2}(t_2)}{\partial B_2(t_2)}\\ \vdots &{} \vdots &{} \vdots &{} \vdots \\ \dfrac{\partial \rho _{m}^{k}(t_i)}{\partial x_{n_m}(t_i)}, &{} \dfrac{\partial \rho _{m}^{k}(t_i)}{\partial y_{n_m}(t_i)}, &{} \dfrac{\partial \rho _{m}^{k}(t_i)}{\partial z_{n_m}(t_i)}, &{} \dfrac{\partial \rho _{m}^{S_k}(t_i)}{\partial B_{n_m}(t_i)} \end{bmatrix}_{n_m\times 4},\\&\mathbf {b_{m}^{ZD}} = \begin{bmatrix} b_{m}^{1}(t_1)\\ b_{m}^{2}(t_2)\\ \vdots \\ b_{m}^{k}(t_i) \end{bmatrix}_{n_m\times 1} \end{aligned}$$

with the index $m= 1,2$ for each telescope. In order to build the single-difference linear equation system from two telescopes to one satellite, we adapt the approach of Beutler et al. (1986, 1987b) to the SLR case using all the available observations at the two telescopes $[\mathbf {b_{1}^{ZD}}, \mathbf {b_{2}^{ZD}}]^T$ which satisfy the quasi-simultaneity condition $\Vert t_i-t_j\Vert \le \delta _t$, and define the single-difference operator $\mathbf {C_{sd}}$ as

$$\begin{aligned} \mathbf {C_{sd}} = \begin{bmatrix} \,1 &{} \,0 &{} \,0 &{} \,\cdots &{} -1 &{} \,0 &{} \,0 &{} \,0\\ \,1 &{} \,0 &{} \,0 &{} \,\cdots &{} \,0 &{} -1 &{} \,0 &{} \,0\\ \,1 &{} \,0 &{} \,0 &{} \,\cdots &{} \,0 &{} \,0 &{} -1 &{} \,0\\ \,0 &{} \,1 &{} \,0 &{} \,\cdots &{} \,0 &{} \,0 &{} -1 &{} \,0\\ \vdots &{} \vdots &{} \vdots &{} \vdots &{} \vdots &{} \vdots &{} \,\cdots &{} \vdots \\ \,0 &{} \,0 &{} \,1 &{} \,\cdots &{} \,0 &{} \,0 &{} \,0 &{} -1\\ \end{bmatrix} \end{aligned}$$

where $C_{sd}$ is a matrix with $n_m$ rows and $2n_m$ columns. The single-difference system is obtained as

$$\begin{aligned} \mathbf {b_{1,2}^{SD}} = \mathbf {C_{sd}}\cdot \begin{bmatrix} \mathbf {b_{1}^{ZD}} \\ \mathbf {b_{2}^{ZD}} \end{bmatrix} \end{aligned}$$

At this point, the assumption of co-located telescopes, separated by a short baseline, comes into play. For short SLR baselines, quasi-simultaneous observations from two telescopes observe the satellite in (approximately) the same relative position, provided that $\delta _t$ is small enough to account for the dynamics of the orbit. Under this assumption of having the same relative geometry for the two telescopes, the partials of the two telescopes become (nearly) identical, this is $\mathbf {A_{1}^{ZD}} = \mathbf {A_{2}^{ZD}}$. Thus, to build the single-difference matrix of partials $\mathbf {A_{1,2}^{SD}}$, only the partials of the first telescope are used. With the help of the single-difference operator, $\mathbf {A_{1,2}^{SD}}$ is calculated as

$$\begin{aligned} \mathbf {A_{1,2}^{SD}} = \mathbf {C_{sd}}\cdot \begin{bmatrix} \mathbf {A_{1}^{ZD}} \\ {\mathbf {0}} \end{bmatrix} \end{aligned}$$

where the zero in the lower part of the matrix represents a matrix with the same dimensions as $\mathbf {A_{2}^{ZD}}$ and whose elements are zero. The $\mathbf {C_{sd}}$ operator is a function of the number of satellites and the number of epochs at which they are observed, which is ultimately defined by the quasi-simultaneity $\delta _t$ used. To take into account the correlations of the single differences, we introduce the weight matrix $\mathbf {P_{sd}}$ (Beutler et al. 1986, 1987b)

$$\begin{aligned} \mathbf {P_{sd}} = \left( \mathbf {C_{sd}}\cdot \mathbf {C_{sd}}^T\right) ^{-1} \end{aligned}$$

**Fig. 2:**
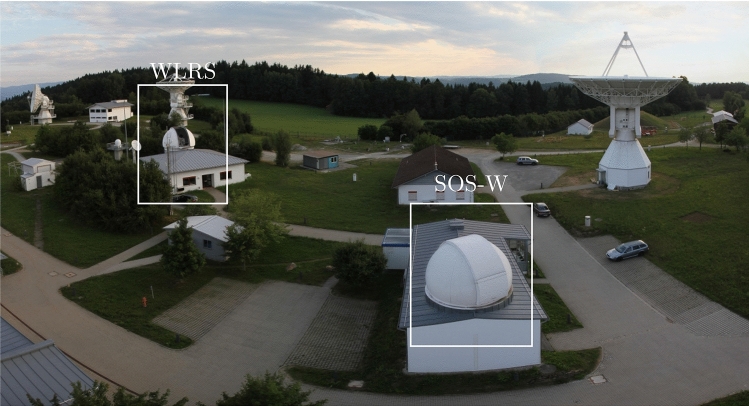
SLR systems at the Geodetic Observatory Wettzell (Source: Technical University Munich)

The solution of the least squares adjustment (LSA) for the single differences, using the matrices $\mathbf {A_{1,2}^{SD}},\ \mathbf {P_{sd}}$ and $\mathbf {b_{1,2}^{SD}}$, provides three relative coordinates ($\Delta X = X_2-X_1,\ \Delta Y = Y_2-Y_1, \Delta Z= Z_2-Z_1$) and the relative range biases $\Delta B$. The quasi-simultaneity $\delta _t$ and, ultimately, the single-differenced linearised equations system are strongly conditioned by the quality and the dynamics of the satellite orbits. For GNSS and LEO satellites this value should not be larger than 2 h, after which it is no longer possible to ensure that satellite positions have a similar error. A detailed analysis of the optimal threshold for the quasi-simultaneity is discussed in Sect. 3.4. With this assumption in mind, single differences of two telescopes to one satellite constitute a tool for the estimation of relative coordinates and range biases differences, while mitigating errors associated to the orbits of the satellites.

### Double-difference observation equations

Similarly to the single-difference case of Sect. 2.2, we build the double-difference observation equation system using all the available observations at the two telescopes $[\mathbf {b_{1}^{ZD}}, \mathbf {b_{2}^{ZD}}]^T$ which satisfy the quasi-simultaneity conditions $\Vert t_{i} - t_{j}\Vert \le \delta _t$ and $\Vert t_{k} - t_{l}\Vert \le \delta _t$, and define the double-difference operator $\mathbf {C_{dd}}$ as 
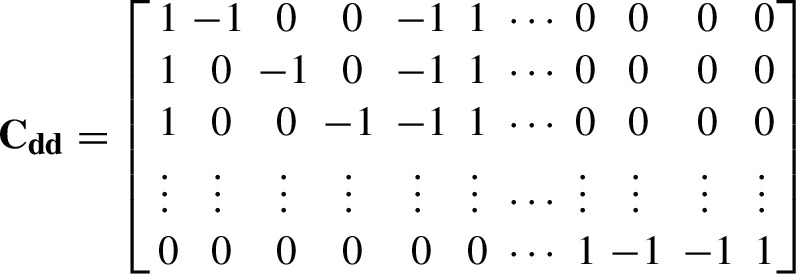
 so that the double-difference system is obtained with

$$\begin{aligned} \mathbf {b_{1,2}^{DD}} = \mathbf {C_{dd}}\cdot \begin{bmatrix} \mathbf {b_{1}^{ZD}} \\ \mathbf {b_{2}^{ZD}} \end{bmatrix}\ \ \ \text {and}\ \ \ \mathbf {A_{1,2}^{DD}} = \mathbf {C_{dd}}\cdot \begin{bmatrix} \mathbf {A_{1}^{ZD}} \\ {\mathbf {0}} \end{bmatrix} \end{aligned}$$

where the zero in the lower part of the matrix represents a matrix with the same dimensions as $\mathbf {A_{2}^{ZD}}$, and whose elements are zero. Notice that this system of linearised equations does not contain the instrumental biases any longer, as they are reduced by the differencing at the same telescope. Similarly to Sect. 2.2, to take into account the correlations between the double differences, the weight matrix $\mathbf {P_{dd}}$

$$\begin{aligned} \mathbf {P_{dd}} = \left( \mathbf {C_{dd}}\cdot \mathbf {C_{dd}}^T\right) ^{-1} \end{aligned}$$

is introduced. The solution of the least squares adjustment for the double differences, using the matrices $\mathbf {A_{1,2}^{DD}},\ \mathbf {P_{dd}}$ and $\mathbf {b_{1,2}^{DD}}$, provides only the three relative coordinates ($\Delta X, \Delta Y, \Delta Z$). To facilitate the understanding of the construction of the single- and double-difference systems of linear equations, Appendix A shows an example of the technical procedure to build these systems, based on real data.

**Table 1:** Coordinates (epoch 2010.0) and velocities of the WLRS system at Wettzell according to  Altamimi et al. (2016). Local tie between WLRS and SOS-W according to Riepl et al. (2019)

**ITRF2014**						
	X [m]	Y [m]	Z [m]	$\mathrm {V_X}$ [m/y]	$\mathrm {V_Y}$ [m/y]	$\mathrm {V_Z}$ [m/y]
**WLRS**	4075576.6506	931785.6790	4801583.6984	−0.0157	0.0171	0.0110
**SOS-W**	4075530.9984	931781.9262	4801619.9971			

**Local Tie**						
$\Delta \mathrm {X}$ [m]	$\Delta \mathrm {Y}$ [m]	$\Delta \mathrm {Z}$ [m]				
45.6522	3.7528	−36.2987				

## Available data and parameterisation

### The co-located SLR telescopes at the Wettzell observatory

The SLR short baseline at the Geodetic Observatory Wettzell is realised by the Wettzell Laser Ranging System (WLRS) and the Satellite Observing System Wettzell (SOS-W), two optical telescopes operating at the wavelengths 532.1 nm and 1064 nm for WLRS and 849.8 nm for SOS-W. While WLRS has been contributing for more than 30 years to the realisation of the ITRF, SOS-W was placed in operation in early 2016 to cope with the need to track more satellites and distribute the workload. The horizontal distance between systems is ca. 58 m, and the difference in height is about 2.3 m (Fig. 2). The local tie vector between the two telescopes has been determined by terrestrial measurements and is continuously compared to the SLR-derived solutions. Moreover, an external calibration target for the SLR systems is available, providing sub-mm accuracy for the definition of the SLR reference points. More details about the operation and the performance of the two systems as well as the characteristics of the local surveys can be found in Riepl et al. (2019). The ITRF2014 coordinates and velocities of the station WLRS from Altamimi et al. (2016) and the local tie to connect WLRS to SOS-W from Riepl et al. (2019) are given in Table 1. Notice that the coordinates of SOS-W are calculated from the ITRF2014 coordinates of WLRS by applying the local tie. More details on the methodology and accuracy of the classical approach for the calculation of the local ties in Wettzell as well as novel approaches for their determination can be found in Klügel et al. (2012); Kodet et al. (2018).

### SLR normal points and meteorological data

The SLR processing using the differencing strategy is based on SLR observations provided as normal point (npt) files. For the scope of this work, we restrict our study to only LAGEOS and GLONASS observations, and we analyse the behaviour of the SLR differences using the available ILRS data (Pearlman et al. 2019b, a; Noll et al. 2019) in the 2-year time interval from 01.01.2018 to 31.12.2019. Selecting those days where WLRS and SOW-S tracked the same satellites, yielded 402 potential sessions with data, from which 172 and 255 sessions contain sufficient GLONASS and LAGEOS data, respectively, to obtain differences. The npt files are converted into range observation files and processed using a dedicated project version of the Bernese GNSS software (BSW) v5.2 (Dach et al. 2015).

**Fig. 3:**
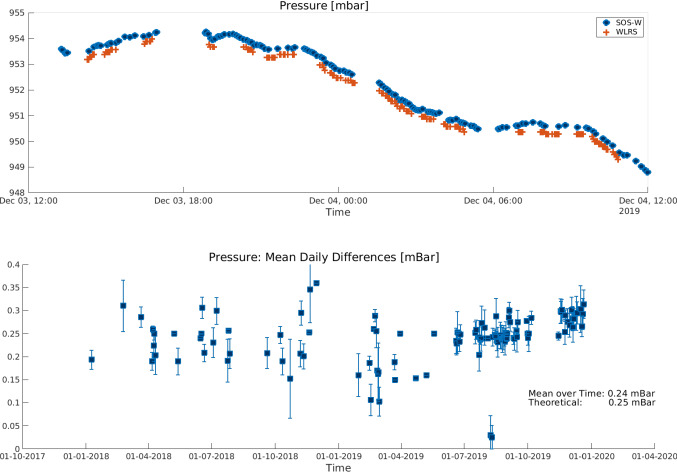
Top: Atmospheric pressure for the two telescopes (WLRS and SOS-W), for days 03.-04.12.2019. Bottom: Mean daily difference (WLRS minus SOS-W) for the linearly interpolated values at common epochs, and their daily standard deviations. The theoretical difference according to Saastamoinen (1972) is given next to the average over the time series

In addition, the meteorological data in the npt files are used to calculate corrections for the range observations associated with the influence of the troposphere, using the standard model of Mendes and Pavlis (2004). One of the main features of the short SLR baseline is the behaviour of the relative troposphere, where for such a small distance and small height change, meteorological data are expected to vary marginally between the two stations. Analogously to GNSS, this guarantees that the dry part of the relative tropospheric delays can be calculated with the use of standard models (Beutler et al. 1987a; Dilßner et al. 2008; Saastamoinen 1972). To test this assumption, the daily values of atmospheric pressure for the two telescopes were interpolated to common epochs, using the nearest neighbour method, and subsequently subtracted, to obtain a daily mean interpolated difference. Figure 3 (top) shows the behaviour of the atmospheric pressure for 03.-04.12.2019 (days of year -DoY- 337 and 338), and the daily mean differences at common interpolated points (bottom), for the 2-year interval. These estimated differences reach 0.26 mbar for the days 03.-04.12.2009 and have an average value of 0.24 mbar over the two investigated years. Additionally, using the height difference between the stations, based on the local tie, a “theoretical” pressure difference was calculated using the simplified model of Saastamoinen (1972) ($P = 1013.25 (1 - 0.0000226 (h-h_0))^{5.225}$). This theoretical difference amounts to 0.25 mbar. The difference between the daily mean and the theoretical value amounts to less than 0.01 mbar, which in turn corresponds to a negligible differential hydrostatic delay in zenith direction (Beutler et al. 1987a). These results support the assumption of a similar troposphere influence on both ends of the short baseline, and they make sure that we do not introduce errors into the baseline results by using erroneous meteorological data.

### Processing strategies: zero-test and baseline estimation

The data analysis is performed with two types of processing strategies. In the first approach, the so-called *zero-test*, coordinates of the stations are derived from the ITRF2014 coordinates and velocities, together with the local tie, and are kept fixed for each processed day. Earth rotation parameters and satellite orbits are assumed fixed and supplied by the products of the Centre for Orbit Determination in Europe (CODE) (Dach et al. 2017), for GNSS, and the ILRS orbits (Pearlman et al. 2019b, a; Noll et al. 2019), for LAGEOS. The meteorological data provided in the npt files are used to correct the ranges for the delays induced by the troposphere, with the help of the empirical model of Mendes and Pavlis (2004). Finally, the range biases were not introduced. With this in mind, each station-satellite range is processed individually at each observation epoch. Under these assumptions, namely fixed station coordinates, orbits and Earth orientation parameters, and fixed troposphere parameters, the zero-test processing of a single station-satellite range does not estimate any geodetic parameters, but it is used to obtain the residual of the measurements with respect to the observation model, which in turn serves as a tool to evaluate the quality and noise of the raw observations. The residuals of the two telescopes, called *zero-difference* residuals, are then subtracted considering the concept of quasi-simultaneity of Sect. 2.1, to build single- and double-differenced residuals.

**Fig. 4:**
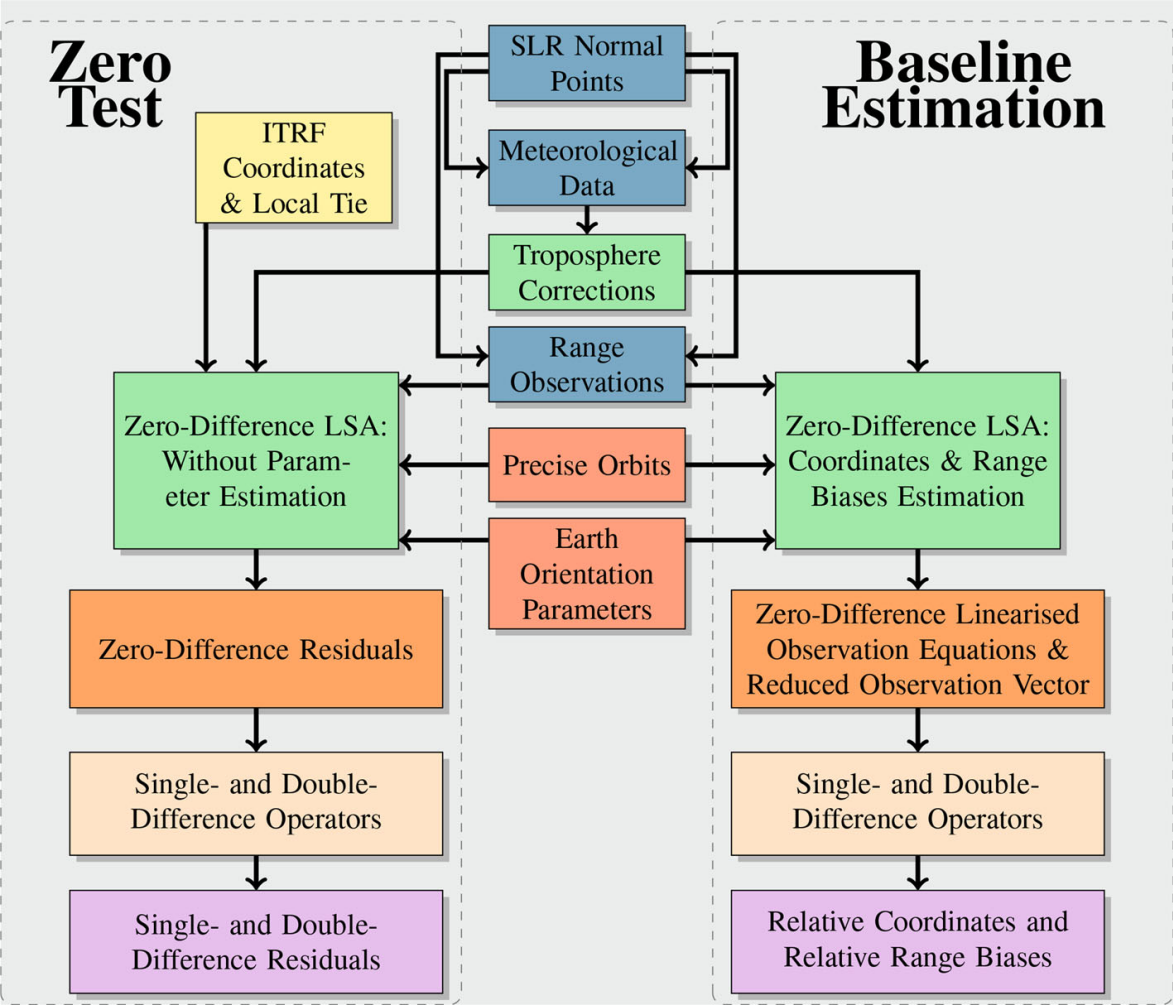
Summary of the investigated processing approaches. While the zero test does not perform the estimation of any parameters, thus delivering only zero-difference residuals wrt. the observation model, the baseline estimation performs the calculation of station coordinates, providing the zero-difference linearised equations and reduced observation vectors

On the other hand, for the *baseline* estimation, Earth rotation parameters, pole coordinates and satellite orbits are once more assumed fixed and supplied by the products (CODE & ILRS), and the meteorological data provided in the npt files are used in the Mendes and Pavlis (2004) model, and again, no range biases were used during the process. In contrast to the zero test, for the baseline estimation, the station coordinates are assumed as unknown and calculated with a weighted least squares adjustment. Once more, each station-satellite range is processed individually, and daily station coordinates of both stations are estimated. This process delivers the zero-differenced linearised equation system $\mathbf {A_{m}^{ZD}}$ and its corresponding zero-differenced reduced observation vector $\mathbf {b_{m}^{ZD}}$, $m=1,2$, for each telescope and each processed day. These linearised equations and reduced observation vectors are then subtracted, using the quasi-simultaneity concept described in Sect. 2.2, to obtain the single- and double-difference linearised equation systems and the single- and double-difference reduced observation vectors, which constitute the main element for our estimation of geodetic parameters. Figure 4 shows a summary description of our two main processing strategies.

Aside from these two differencing strategies, “standard” SLR solutions are calculated. Within these solutions coordinates and range biases per constellation are determined to benchmark the results of the differencing methods. Their daily normal equations are stored and combined to obtain parameters with the same validity as those obtained with the differencing approaches.

**Fig. 5:**
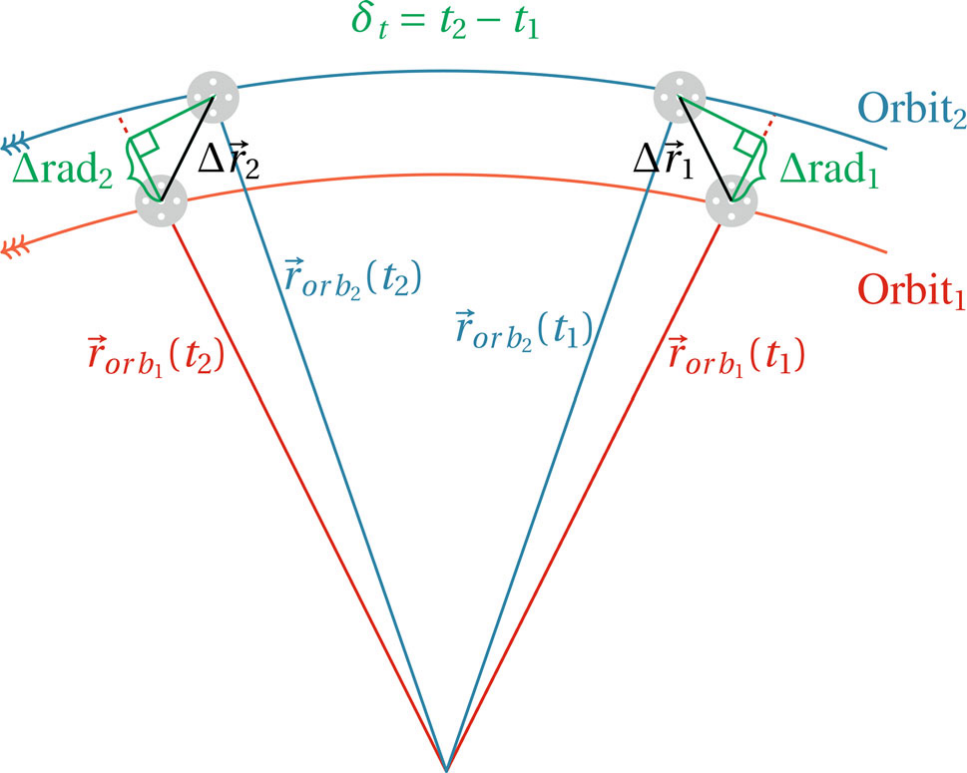
Concept for the determination of the best quasi-simultaneous threshold. Using only one satellite, two different orbit solutions (from different processing centres) are compared. First, perfectly simultaneous single differences between orbit solutions are formed ($\Delta \mathbf {r}_1$ and $\Delta \mathbf {r}_2$, with their corresponding radial components $\Delta \text {rad}_1$ and $\Delta \text {rad}_2$). Then, double differences with all possible time steps are built, and the RMS value of the double differences at a certain time step is calculated

**Fig. 6:**
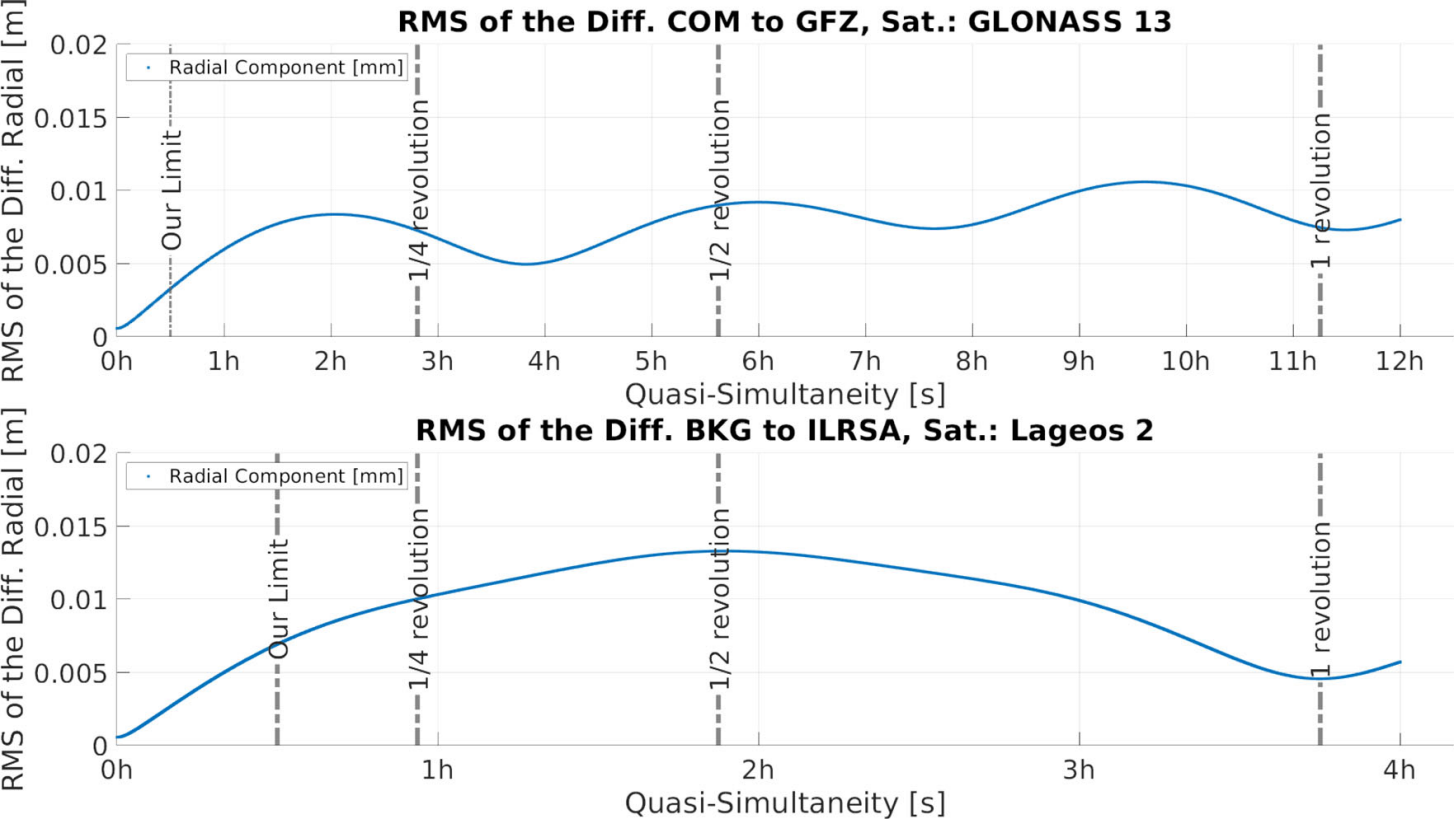
RMS of the double differences to a single satellite, in relation to the quasi-simultaneity, for the radial component. Top: Satellite GLONASS 13. Bottom: Satellite LAGEOS 2. These differences have been built using two different orbital products. Moreover, the bins for the RMS calculation are considered with quasi-simultaneity $QS = d_t$ instead of $QS \le d_t$

**Fig. 7:**
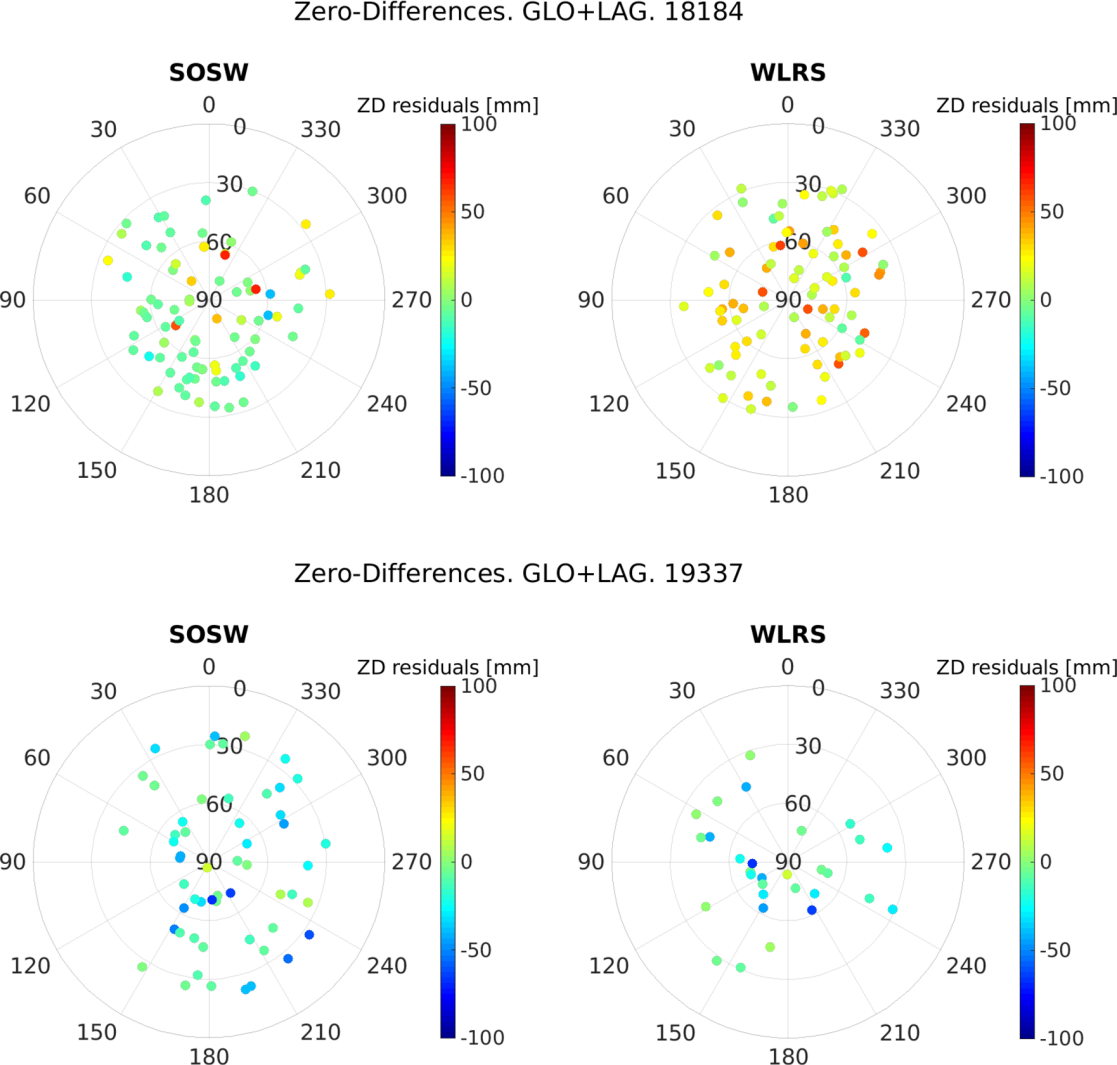
Skyplots of the zero-difference residuals of the zero test, separately for each telescope. Top: 03.07.2018 (DoY 184, 2018). Bottom: 03.12.2019 (DoY 337, 2019)

### Selection of the threshold for the quasi-simultaneity

As mentioned before, the threshold for the quasi-simultaneity $\delta _t$ is conditioned by the quality and the dynamics of the satellite orbits. To determine the impact of this limit on the double differences, we designed a test using satellite orbit solutions from two different processing centres, reflecting the orbital errors to be expected, to form single and double differences. The solutions involved are COM (Center for Orbit Determination in Europe -CODE-) and GFZ (GeoForschungsZentrum Potsdam) for the GLONASS satellites, and ILRSA (International Laser Ranging Service) and BKG (Bundesamt fuer Kartographie und Geodäsie) for the LAGEOS satellites.

To simulate the effect of the orbital errors on the double-difference observations, we form the double differences of the two orbits according to Fig. 5 and the formula

$$\begin{aligned}&\displaystyle \mathbf {r}_{DD}(\delta _t = t_2-t_1) = \Delta \mathbf {r}_{2} - \Delta \mathbf {r}_{1} {=} \left( \mathbf {r}_{orb_2}(t_2) {-} \mathbf {r}_{orb_1}(t_2)\right) \\&\quad - \left( \mathbf {r}_{orb_2}(t_1) - \mathbf {r}_{orb_1}(t_1)\right) \end{aligned}$$

Thereby $\delta _t = t_2-t_1$ denotes the quasi-simultaneity value of the double-difference orbit errors. Using values of 1 s, 2 s,..., for the quasi-simultaneity we can compute the RMS of the radial orbit error double differences as a function of $\delta _t$. This procedure is done individually for each satellite, and we use “equal” instead of “smaller than” for the quasi-simultaneity. Of special interest is the radial component of the orbit, as it is closely related to the SLR ranges. As we perform the differences with *exactly* certain time step ($QS = \delta _t$), instead of less than or equal to ($QS \le \delta _t$), the number of differences decreases when increasing the lapse for the difference. From each of these groups of differences (with quasi-simultaneity 1 s, 2 s, ...) we take the RMS and use it as a metric for our analysis.

Figure 6 shows these RMS values, for the radial component, in relation to the quasi-simultaneity, where the top plot shows the RMS values at each quasi-simultaneity for satellite GLONASS 13 and the bottom plot for satellite LAGEOS 2. In both cases the quasi-simultaneity was truncated at about the revolution period of each satellite: 223 min for LAGEOS 2 and 11 h 15 min for GLONASS 13. As expected, the RMS increases with the quasi-simultaneity. Moreover, it is evident that if all differences smaller or equal to a certain quasi-simultaneity were considered, the RMS values would grow in a similar way. It is also visible that a threshold for the quasi-simultaneity of 0.5 h ensures a relatively small RMS value. In this way we guarantee that possible errors of the orbits are not propagating into the differences.

## Results

### Analysis of the residuals from the zero test

With the zero-test strategy described in Sect. 3.3, the SLR data corresponding to the two telescopes in the time interval between 01.01.2018 and 31.12.2019 were processed in daily sessions, using the BSW software. Figure 7 shows the behaviour of the GLONASS and LAGEOS residuals as a function of the azimuth and elevation angle, for two of these days, namely 07.03.2018 (2018, DoY 184) and 03.12.2019 (2019, DoY 337), displayed by telescope. These residuals represent the level of noise of the observations plus orbit errors and possible instrumental biases. A summary of the statistics of these residuals is shown in Table 2. Aside from the difference in the number of observations and the presence of few outliers, the residuals of both days are among nominal values for the SLR technique, between $-10$ cm and 10 cm. However, there is a large contrast between the mean values obtained for the data set on the left wrt. the data set on the right in Table 2, mainly caused by the range bias of the station.

**Table 2:** Summary statistics of the zero-difference residuals of the zero test, separately for each telescope. Left: 03.07.2018 (DoY 184, 2018). Right: 03.12.2019 (DoY 337, 2019)

		Mean [mm]	Std [mm]
Data from 03.07.2018			
SOS-W	$\text {GLO}$	1.5	11.7
	$\text {LAG}$	6.9	2.6
WLRS	$\text {GLO}$	21.6	9.7
	$\text {LAG}$	7.8	6.0
Data from 03.12.2019			
SOS-W	$\text {GLO}$	−11.1	7.0
	$\text {LAG}$	−12.3	7.9
WLRS	$\text {GLO}$	−14.9	11.4
	$\text {LAG}$	−8.4	7.9

**Fig. 8:**
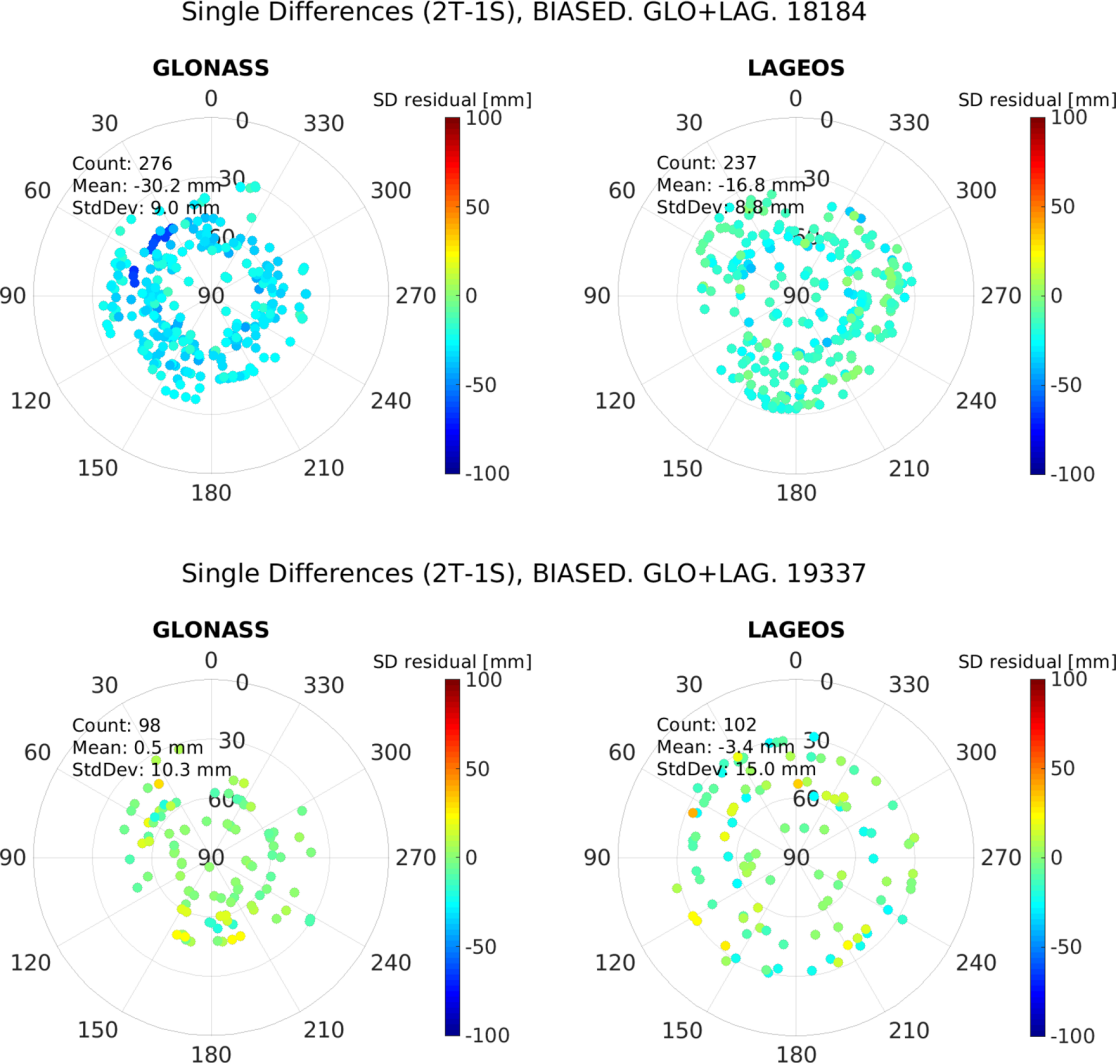
Skyplots of the single-difference residuals of the zero test, using a quasi-simultaneity of 2 h, separately for each constellation. Top: 03.07.2018 (DoY 184, 2018). Bottom: 03.12.2019 (DoY 337, 2019)

**Fig. 9:**
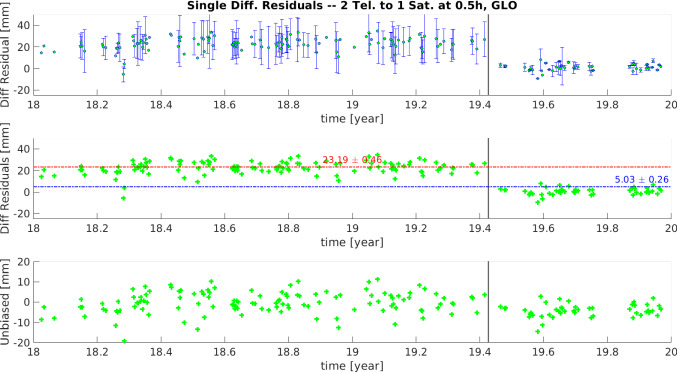
Time series of the daily mean single-difference residuals of the zero test, at quasi-simultaneity of 0.5 h, for the GLONASS satellites. Top: Mean daily single difference at quasi-simultaneity 0.5 h, with its corresponding daily standard deviation. Middle: Mean daily single difference at quasi-simultaneity 0.5 h, averaged over the two years to obtain the (relative) range biases. Bottom: Unbiased time series of daily mean single differences, after removing the range biases

**Fig. 10:**
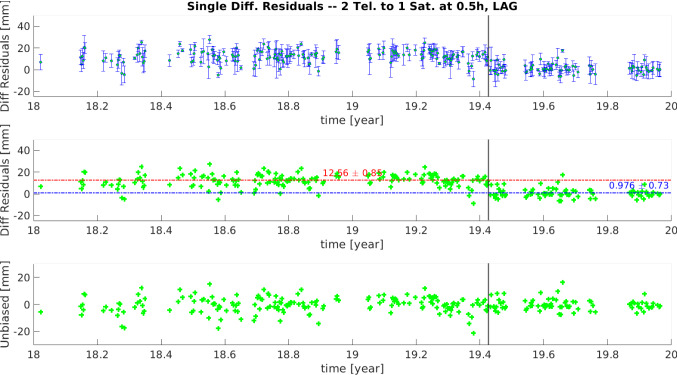
Time series of mean single-difference residuals of the zero test, at quasi-simultaneity of 0.5 h, for the LAGEOS satellites. Top: Mean daily single difference at quasi-simultaneity 0.5 h, with its corresponding daily standard deviation. Middle: Mean daily single difference at quasi-simultaneity 0.5 h, averaged over the two years to obtain the (relative) range biases. Bottom: Unbiased time series of daily mean single differences, after removing the range biases

To further investigate the presence of instrumental biases associated with the stations, the single difference of the zero-difference residuals is built. For this, we considered a quasi-simultaneity of 2 h, and built the differences from two telescopes to one satellite. For the 03.07.2018, 513 single-difference residuals are formed, from which 276 correspond to differences to GLONASS satellites, and the remaining 237 are to LAGEOS. The same procedure produced 200 single-difference residuals for day 03.12.2019 with 98 differences for GLONASS and 102 for LAGEOS. These difference residuals are displayed in Fig. 8. As the range bias for the WLRS station was not applied during the processing, the single-difference residuals of day 03.07.2018 show a distinctive bias with a mean of $-30.2$ mm and $-16.8$ mm for GLONASS and LAGEOS, respectively. However, the difference residuals of the second data set are almost unbiased, with mean bias values of 0.5 mm and −3.4 mm for GLONASS and LAGEOS, respectively. This difference in bias results might be due to the different target response of the two SLR systems wrt. the detectors.

**Fig. 11:**
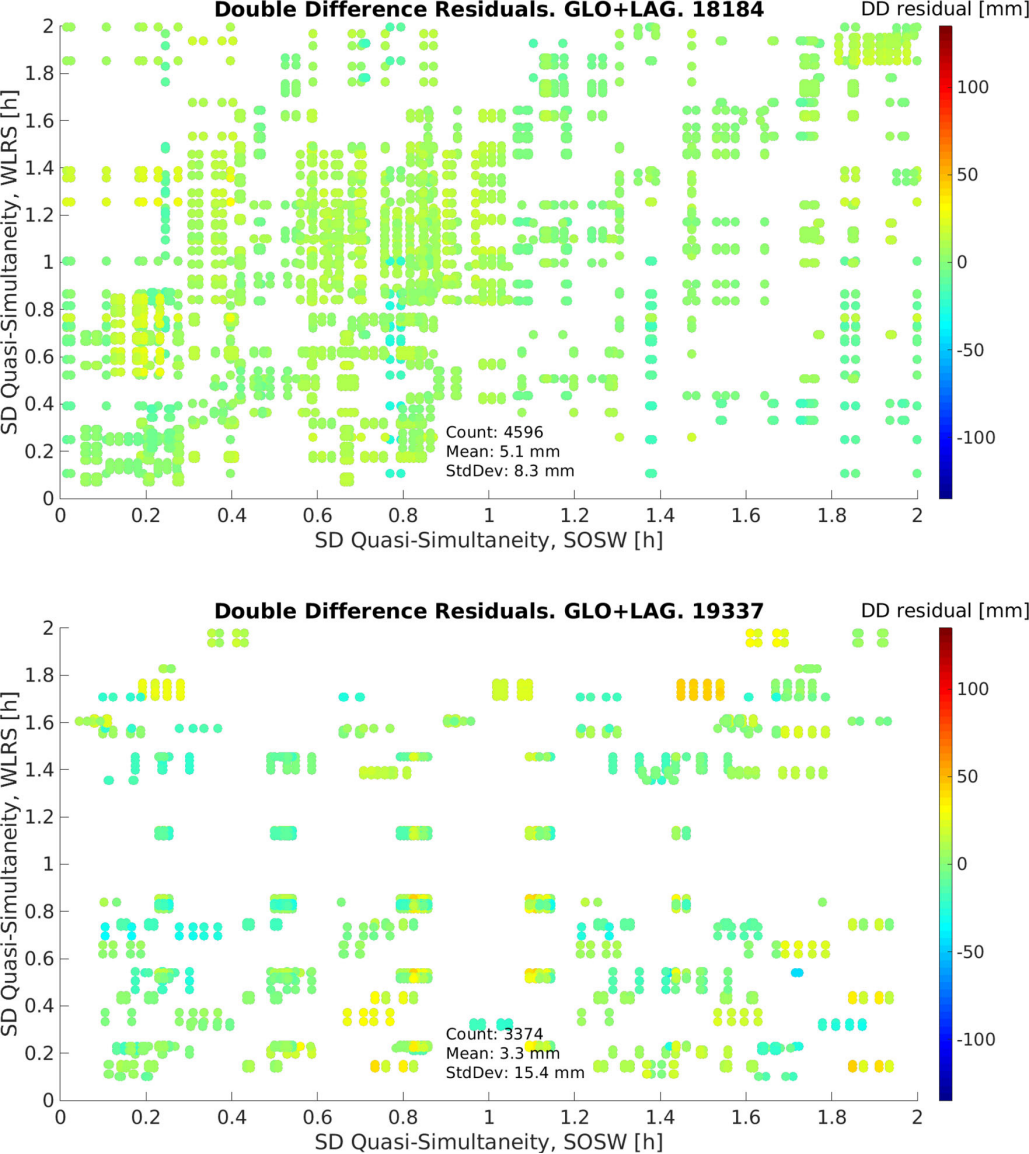
Double-difference residuals of the zero test, as a function of the quasi-simultaneity up to 2 h, for both, the WLRS and SOS-W telescope. Top: 03.07.2018 (DoY 184, 2018). Bottom: 03.12.2019 (DoY 337, 2019)

**Fig. 12:**
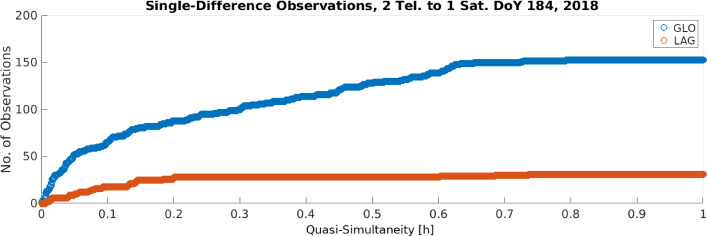
Available single-difference observations, as a function of the quasi-simultaneity up to 1 h, separately for each constellation, for the day 03.07.2018 (DoY 184, 2018)

**Fig. 13:**
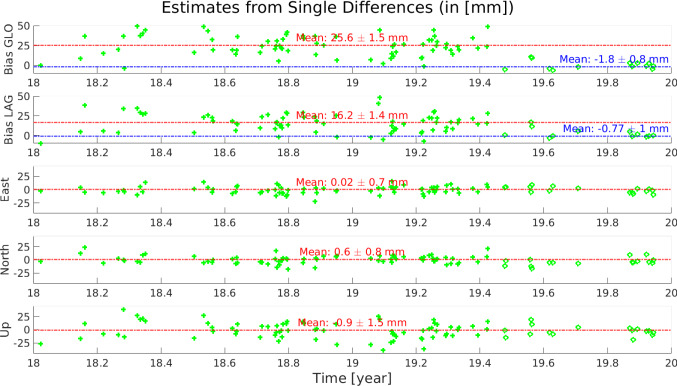
Time series of daily estimated parameters using single differences, when selecting as representative of each day the values obtained with a quasi-simultaneity of 0.5 h. The ENU fields correspond to the differences of the estimated values wrt. the local ties

To determine the behaviour of the instrumental bias and establish the time of possible bias changes, the time series of single-difference residuals is analysed. As seen in Fig. 8, when using a quasi-simultaneity of 2 h, it is possible to build multiple single differences per day. In light of the results of Sect. 3.4, where a threshold of quasi-simultaneity of 0.5 h was found as the best option to minimise errors from the orbits, we consider as unique “representative” for each day the differences with quasi-simultaneity $\le$ 0.5 h. These daily values are displayed in the top plots of Figs. 9 and 10, where the mean single-difference residuals, together with the daily standard deviation, are displayed. While the standard deviation of the daily mean difference residuals is considerably larger for the GLONASS satellites than for LAGEOS, both time series display a distinctive break ca. 01.06.2019, confirming the results seen in Fig. 8. Therefore, we calculated the weighted mean value of these daily results imposing a break on DoY 156 of 2019, and the corresponding error for the mean is calculated. The middle plots of Figs. 9 and 10, show these mean values over the entire time series, where the vertical black line indicates the date of the break imposed for the calculation. Notice that the imposed break ensures the calculation of different values for the instrumental biases, 23.19 mm and 5.03 mm for GLONASS, and 12.56 mm and 0.98 mm for LAGEOS, with sub-mm errors for these mean values. If these instrumental biases are removed, the (unbiased) time series show a zero-mean behaviour (bottom plots of Figs. 9 and 10). These results demonstrate that the use of SLR single differences can be a useful tool for the accurate estimation of instrumental biases.

When now forming the double-difference residuals, the effects of these instrumental biases, and possible errors in the orbits of the satellites, are eliminated or mitigated. Figure 11 shows the double-difference residuals of days 03.07.2018 and 03.12.2019 allowing a quasi-simultaneity of 2 h. In these figures, the x-axis indicates the quasi-simultaneity allowed building the single-differenced residuals from the SOS-W telescope to two satellites, while the y-axis shows the quasi-simultaneity allowed to build the single differences from the WLRS telescope to two satellites. Finally, the colour bar indicates the value of the residuals. With this approach we built 4596 and 3374 double differences, for day 03.07.2018 and 03.12.2019, respectively. These residuals are no longer biased, with mean values of 5.1 mm for the former and 3.3 mm for the latter day. These small mean daily values, together with mm-level standard deviations, are an indication that SLR double-difference observations are potential candidates for the estimation of geodetic parameters.

**Fig. 14:**
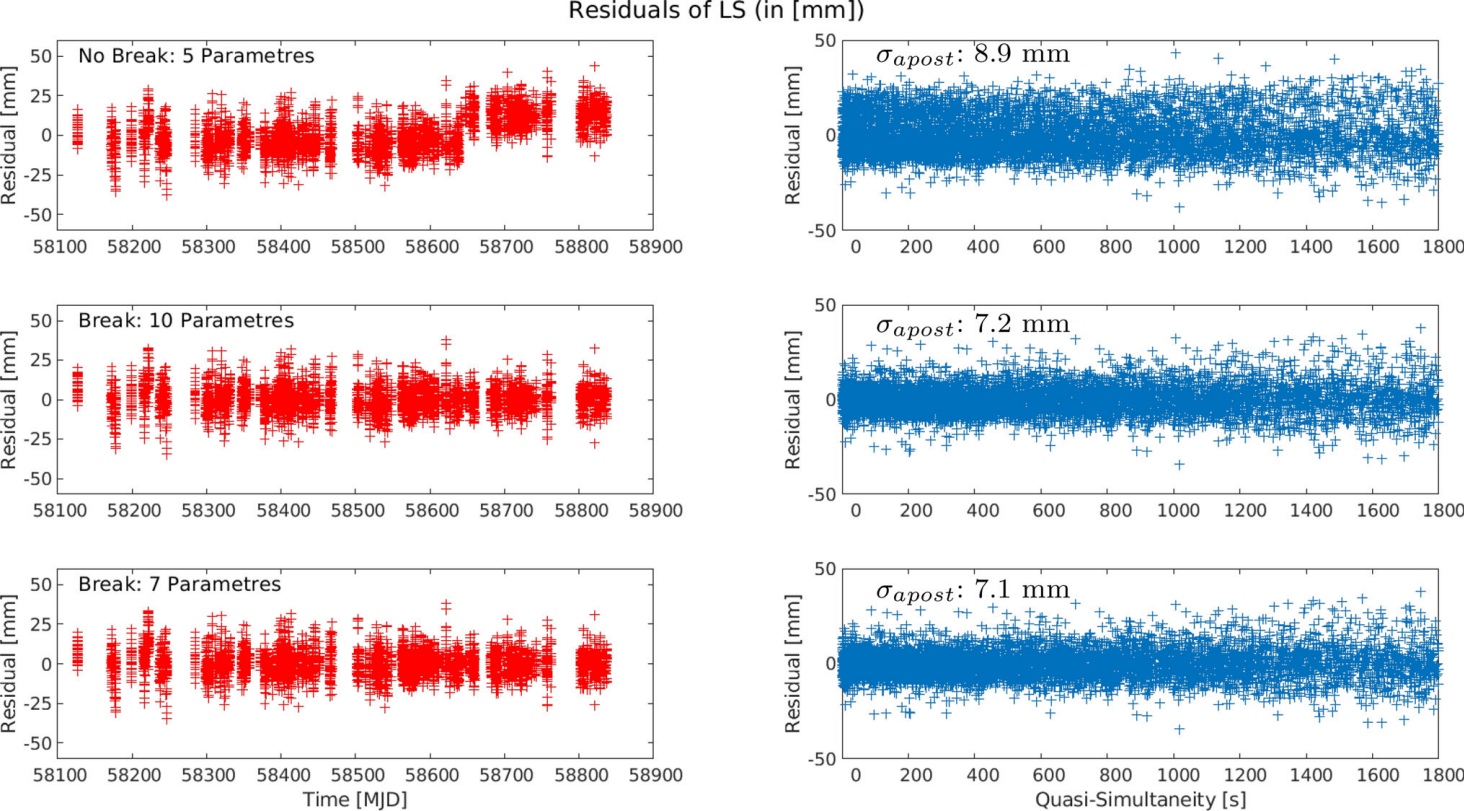
Residuals of the unified weighted least squares adjustment, for the single differences case. Left: Time series with respect to the day of observation (mean Julian date). Right: Residuals in relation to their quasi-simultaneity and their *sigma*-aposteriori $\left( \frac{v^TPv}{n-u}\right)$

### Baseline estimation based on single differences

The first step of the baseline estimation process performs the determination of coordinates and instrumental biases based on single differences from two telescopes to one satellite, for each day individually. All the linearised equations satisfying the quasi-simultaneity condition $\Vert t_i -t_j\Vert \le \delta _t$ with $\delta _t =0.5$ hours, are stacked, as described in Sect. 2.2, and a weighted least squares adjustment is used to derive the corresponding 5 parameters, three relative coordinates ($\Delta X,\ \Delta Y, \Delta Z$) and two range biases, one for GLONASS and one for LAGEOS. For instance, the behaviour of the aggregated instrumental bias at the day 03.07.2018, shows the trade-off between a larger quasi-simultaneity value and the orbital errors, a crucial point at this stage of the estimation. While large values of quasi-simultaneity guarantee a larger number of observations, which in turn favour the estimation process and improve the formal errors, the errors caused by the orbits (intrinsic to our method) grow larger. Due to the large amount of data available on day 03.07.2018 (Fig. 12), the values at quasi-simultaneity 0.5 h, $21.9\pm 2.3$ mm for GLONASS and $14.8\pm 2.1$ for LAGEOS, are in agreement with the preliminary values observed for the same day in Sect. 4.1, during the entire session. Similarly, the difference of the estimates for the east, north, and up (ENU) components of the baseline with respect to the local tie, shows that the daily estimated values are in a close agreement with the local tie, with mean differences per component of $[4.7,\ 4.5,\ -10.1]\ \pm \ [1.2,\ 0.9,\ 2.9]$ mm, for east, north, and up, respectively. As a compromise between the number of observations and the influence of the orbital errors, we have selected those values obtained for a quasi-simultaneity of 0.5 h for each day’s weighted least squares estimation. With this, we form the time series of daily estimates, three coordinate components, and two biases (GLONASS and LAGEOS), over the period of the two years of interest. Figure 13 shows these time series, with the time (year) of observation in the x-axis. This daily estimation additionally shows the need to differentiate between the instrumental biases before and after the observed break at day 01.06.2019.

**Table 3:**
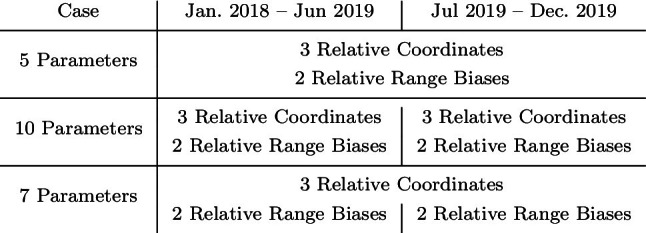
Timeline of the validity of the parameters estimated with the three rigorous LSA, based on single-difference observations

These results support the results of Sect. 4.1 regarding the changes in the instrumental biases and the need for a break in the data during the estimation process. For the coordinate estimates, the differences between the ENU estimates and the local ties are displayed. This daily analysis also facilitates the assessment of the performance of the differencing strategies regarding the number of formed differences and possible outliers.

**Table 4:** Differences of the ENU estimates wrt. the local tie and formal errors of the rigorous two-year (unified) weighted least squares adjustment, based on single differences from two telescopes to one satellite. All values in millimetres

	Without break (5 Param.)		With break (10 Param.)		With break (7 Param.)	
	Estimate	Formal Error	Estimate	Formal error	Estimate	Formal error
E [mm]	1.1	1.0	0.4	0.6	0.2	0.7
			−0.5	0.3		
N [mm]	0.6	1.0	1.6	0.5	0.8	0.6
			−1.8	0.3		
U [mm]	−0.7	1.1	−1.9	0.6	−2.1	0.7
			−2.0	0.4		
Bias GLO [mm]	20.8	0.9	24.3	0.5	24.0	0.5
			0.1	0.2	0.4	0.2
Bias LAG [mm]	11.6	1.5	14.9	0.8	14.8	0.8
			−1.3	0.5	−1.3	0.5

**Fig. 15:**
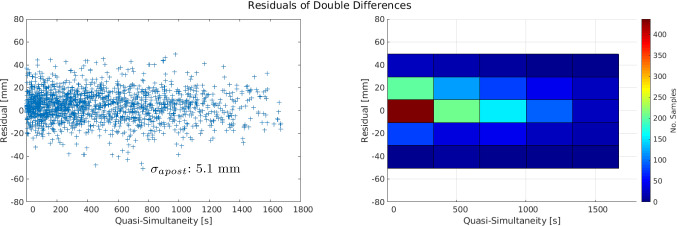
Residuals of the rigorous (unified) weighted least squares adjustment, for the double-differences case. Left: Residuals in relation to their quasi-simultaneity, with their $\sigma$-aposteriori. Right: Histogram of residuals in relation to their quasi-simultaneity

**Fig. 16:**
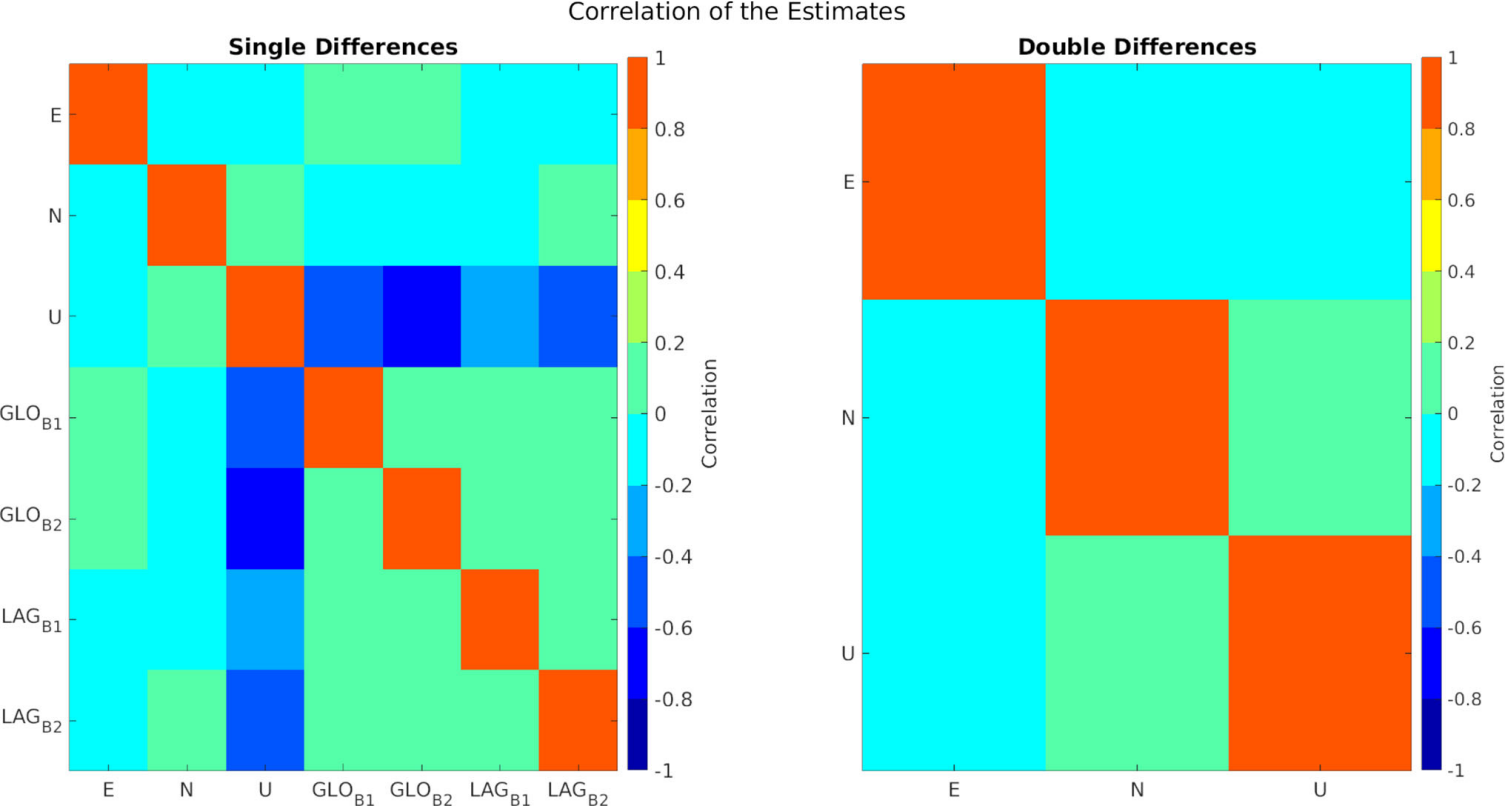
Correlations between estimated parameters for two of the investigated approaches. Left: Based on single differences with 7 parameters. Right: Based on double differences

Despite the high level of agreement between the daily estimates and the local tie, for a more rigorous and accurate solution, a complete (unified) weighted least squares solution is preferred. This least squares solution is computed by stacking all the daily linearised equations with quasi-simultaneity $\le$ 0.5 h over the two years and producing a unique weighted least squares solution. When considering this approach with the available data, three cases are possible (Table 3):

1. Estimation of five parameters, three coordinates, and two range biases, where jump in the biases is not considered
2. Estimation of ten parameters, three coordinates, and two range biases before and the same 5 parameters after the observed bias jump
3. Estimation of seven parameters, three coordinates, and four range biases. These four biases correspond to two biases for GLONASS and two for LAGEOS, when considering the bias jump in the observation time series

Figure 14 shows the residuals of the weighted least squares adjustment for these three cases. The time series with respect to the day of observation, shows an evident break for the residuals when estimating only five parameters (plot on the top-left), while the cases where breaks in the instrumental biases were considered, show unbiased zero-mean residuals in time. The right plot of Fig. 14 also shows that for larger values of quasi-simultaneity the residuals of the estimation begin to slowly increase, justifying the selection of a relatively small quasi-simultaneity value of 0.5 h.

Table 4 summarises the estimated values, together with the formal errors, of these three cases. As done before, the ENU estimates were subtracted from the local tie, so the values depict the difference of the SLR estimates with respect to the terrestrial measurements. While all the coordinate estimates are in a millimetre to sub-millimetre agreement with the local ties, there is an improvement in the formal errors for those solutions considering a break for the instrumental biases in the data. A tendency of lower values of the formal errors in the north component is seen in the three approaches, as a result of the south–north orientation of the baseline. As in the case of the GNSS-based solutions, the formal errors of the up component show the largest values. When comparing the values for the estimates of the instrumental biases, we see a clear difference between the approach with only five parameters and the remaining two cases. Looking at the solutions considering breaks for the range biases, we see that the biases for the GLONASS satellites change from 24.3 mm to 0.1 mm and 24.0 mm to 0.4 mm, for the 10 and 7 parameters solutions, respectively. These changes are statistically significant, when considering the formal errors of these values: 0.49 mm and 0.23 mm for the 10 parameters, and 0.50 mm and 0.23 mm for the 7 parameters. Similarly, with the formal errors found for the range biases, the change in the range biases is statistically significant and cannot be ignored. These results support the preference for the solution including a break for the estimation of the instrumental biases. As the coordinates of the stations are not expected to change due to the change in the bias, the solution with 7 parameters, where the components of the baseline vector are estimated once, is preferred. The correlations among the estimates for this approach are shown in the left part of Fig. 16. The small correlations ($\Vert \rho \Vert \le .25$) among the coordinate components, zero correlation among the biases, and the expected large correlations between the height component and the range biases, support this selection.

### Baseline estimation based on double differences

Although the estimated coordinates based on single differences from Sect. 4.2 show a millimetre agreement with the local tie, with relatively low formal errors, possible temporal variations of the instrumental biases are still present in the adjustment and may have an impact on the final solution. To avoid these issues with the determination of instrumental biases and to reduce and mitigate the influence of other error sources, a double-difference weighted least squares estimation has been performed. Moreover, as discussed in Sect. 3.2, due to the short distance between the two telescopes, the influence of the troposphere on the SLR signal is expected to be the same (apart from the height difference). Therefore, with the use of double-difference observations, the tropospheric delay affecting the original observations is mitigated or strongly reduced.

To perform the estimation process, we used the idea of Sect. 2.3 to select the daily linearised equations which satisfy the quasi-simultaneity condition $\delta _t\le 0.5$ h. This set of linearised equations is free of the influence of the troposphere, instrumental biases, and with a reduced influence of orbital errors. Instead of performing a daily estimation of parameters and then a calculation of the corresponding mean, we stack the linearised equations for one rigorous 2-year (unified) weighted least squares adjustment, where the only unknowns are the components of the baseline between the telescopes. Figure 15 shows the behaviour and distribution of the residuals of this weighted least squares adjustment, where the absence of any systematic influence of the instrumental biases is noticed. Moreover, for relatively short periods of quasi-simultaneity ($\le 600$ s) the majority of the residuals of the double differences are between -10 mm and 10 mm. As for the single-difference case, we calculate the differences between the resulting coordinates and the local tie. The ENU components of this difference amount to:

$$\begin{aligned}&\mathrm {East:}\ 0.7 \pm 0.2~\mathrm {mm},\ \mathrm {North:}\ -0.9 \pm 0.2~\mathrm {mm},\ \mathrm {Up:}\\&\quad -0.6\pm 0.2~\mathrm {mm} \end{aligned}$$

Furthermore, the correlations among these estimates (right part of Figure 16) show small values. The high level of agreement with the local tie and favourable correlations among the estimates, support the use of SLR double differences for the estimation of the local short baseline.

**Table 5:** Estimated ENU coordinate difference w.r.t the local ties, and instrumental biases with the different strategies discussed in this paper. The left column shows the value for the estimated parameter, and the right column the corresponding formal error. All values in millimetres

							Differencing approaches											
	Bernese zero estimation						Fixed to		Single Differences								Double	
	(Difference of Estimates)						Local Tie		2 Telescopes - 1 Satellite								Differences	
	5 Estimates		10 Estimates		7 Estimates		Only Biases		Mean of Daily		5 Estimates		10 Estimates		7 Estimates		3 Estimates	
	(No Break)				(4 Biases)		(4 Par.)		Estimates (5 Par.)		(No Break)				(4 Biases)		(No Biases)	
	Param	$\sigma$	Param	$\sigma$	Param	$\sigma$	Param	$\sigma$	Param	$\sigma$	Param	$\sigma$	Param	$\sigma$	Param	$\sigma$	Param	$\sigma$
E [mm]	0.4	0.9	0.3	1.0	0.2	0.7			−0.5	0.6	1.1	1.0	0.4	0.6	0.2	0.7	0.7	0.2
			0.2	1.9									−0.5	0.3				
N [mm]	−0.5	1.7	1.5	2.0	−0.1	1.3			0.1	0.9	0.6	1.0	1.6	0.5	0.8	0.6	−0.9	0.2
			−5.0	3.6									−1.8	0.3				
U [mm]	10.2	2.2	8.0	3.6	10.5	1.7			−0.5	1.4	−0.7	1.1	−1.9	0.6	−2.1	0.7	−0.6	0.2
			−17.3	4.6									−2.0	0.4				
$\mathrm {GLO}_b$ [mm]	37.3	1.9	39.6	2.2	41.2	1.5	23.1	0.5	25.6	1.5	20.8	0.9	24.3	0.5	24.0	0.5		
			22.2	4.0	18.2	1.7	5.3	0.3	−1.8	0.8			0.1	0.2	0.4	0.2		
$\mathrm {LAG}_b$ [mm]	19.4	1.8	21.5	2.1	23.0	1.4	12.6	0.9	16.2	1.4	11.6	1.5	14.9	0.8	14.8	0.8		
			14.3	3.8	9.6	1.6	1.0	0.7	−0.7	1.0			−1.3	0.5	−1.3	0.5		
											Stacking of linearised equations (“Rigorous 2-years LSA”)							

## Conclusions

The single- and double differences of SLR observations for the short baseline in Wettzell have been investigated, and a novel approach to build differences of SLR observations has been developed, based on the concept of quasi-simultaneity. These differences are built with the linearised equations of the zero-difference processing. Therefore, the interpolation of the SLR ranges is no longer required, and systematic errors common to the baseline can be targeted. The experiments over two years of SLR observations with the co-located telescopes in Wettzell, showed the advantages of the proposed method, namely the estimation of relative coordinates, suitable for the validation of local ties, and the estimation/elimination of instrumental range biases. Table 5 summarises the estimated values obtained using all the investigated methods, in addition to the “standard” results obtain for the same time interval (Bernese Zero Estimates). In particular, the single-difference rigorous solution with 7 estimates (for 3 relative coordinates and 2 relative range biases for LAGEOS and GLONASS) shows a much higher level of agreement with respect to the local ties, especially in the up component, with a difference of -2.1 mm against the 10.5 mm of the standard zero-difference solution. Furthermore, the formal errors of the ENU components for the single-difference method are significantly better than those of the zero differences. While there is no benchmark for the values of the range biases, the analysis of the formal errors of the single differences in relation to the standard method of the zero differences, shows a much higher performance of the former, with a sub-mm level for the errors. However, in contrast to the zero-difference approach, the single-difference strategy is only able to provide range bias differences between the stations. Nevertheless, to avoid the estimation of range biases, the double differences were studied. With the elimination of these biases, the estimation of relative coordinates, and therefore the validation of the local ties, is performed clearly more accurately. We found that the agreement of the relative coordinates and the local tie is within 1 mm for each of the ENU components, with corresponding formal errors in the sub-mm domain. Moreover, with a $\sigma _{apost}$ of 5.1 mm of the double-difference solution, against 7.1 mm for the solution based on single differences, the double-difference approach shows an additional and more effective reduction of systematic biases. The main improvement is observed in height component, which corresponds to the expectations, as the error sources mitigated with our approach (range biases, orbit errors, troposphere) are influencing the height the most. These characteristics support the usability of the proposed differencing methods for the estimation of geodetic parameters with a high degree of accuracy and their usability for the validation of local ties. Future activities will include the study of longer baselines, where we expect that the principle still works, however with a less pronounced reduction of orbit and tropospheric errors.

## Data Availability

The SLR data sets used for this study are available from ftp://edc.dgfi.tum.de/pub/slr/data. The GNSS orbits used can be found at ftp.aiub.unibe.ch/CODE_MGEX/CODE/, and the LAGEOS orbits can be found at ftp://edc.dgfi.tum.de/slr/products/orbits/. The data sets generated during this study are available from the corresponding author on reasonable request.

## Appendix: single- and double-difference systems of linear equations

Table 6 shows a sample of observations collected at the two telescopes in Wettzell for DoY 184, 2018.

**Table 6:** Linearised observation equations obtained when processing the raw SLR ranges (zero differences) for telescopes SOS-W and WLRS

Telescope	Sat.	MJD	$\mathbf {A^{ZD}_m}$			$\mathbf {b^{ZD}_m}$
SOS-W	952	58302.14577	−0.9341	0.3497	−0.0714	0.0285
SOS-W	104	58302.12057	−0.9269	−0.3715	−0.0532	0.0061
SOS-W	952	58302.14662	−0.9400	0.3216	−0.1137	0.0075
SOS-W	119	58302.29856	−0.7634	−0.6091	−0.2149	0.0093
SOS-W	109	58302.29092	−0.3450	0.1958	−0.9179	0.0053
WLRS	952	58302.16049	−0.6007	−0.3553	−0.7162	−0.0080
WLRS	104	58302.11941	−0.9258	−0.3715	−0.0702	−0.0216
WLRS	952	58302.16049	−0.6007	−0.3553	−0.7162	−0.0080
WLRS	119	58302.30681	−0.7328	−0.5934	−0.3330	−0.0289
WLRS	109	58302.27069	−0.3365	0.4532	−0.8255	−0.0348

Considering a quasi-simultaneity condition of $\delta _t \le 1~h$, the single differences are built with the help of the $\mathbf {C_{sd}}$ operator

Similarly, the double differences are built using the $\mathbf {C_{dd}}$ operator

Thus, the single- and double-differenced linear equation systems are given by

$$\begin{aligned} \mathbf {b_{1,2}^{SD}}= & {} \mathbf {C_{sd}}\cdot \mathbf {b^{ZD}_{1}}\ \ \ \text {and}\ \ \ \mathbf {A_{1,2}^{SD}} = \mathbf {C_{sd}}\cdot \begin{bmatrix}\mathbf {A^{ZD}_{1}}\\ \mathbf {0_{5\times 3}}\end{bmatrix}\\ \mathbf {b_{1,2}^{DD}}= & {} \mathbf {C_{dd}}\cdot \mathbf {b^{ZD}_{1}}\ \ \ \text {and}\ \ \ \mathbf {A_{1,2}^{DD}} = \mathbf {C_{dd}}\cdot \begin{bmatrix}\mathbf {A^{ZD}_{1}}\\ \mathbf {0_{5\times 3}}\end{bmatrix}\\ \end{aligned}$$
